# Epidemiologic Behavior of Obesity in the Maracaibo City Metabolic Syndrome Prevalence Study

**DOI:** 10.1371/journal.pone.0035392

**Published:** 2012-04-18

**Authors:** Valmore Bermúdez, Maikol Pacheco, Joselyn Rojas, Evelyn Córdova, Rossibel Velázquez, Daniela Carrillo, María G. Parra, Alexandra Toledo, Roberto Añez, Eneida Fonseca, Rafael París Marcano, Clímaco Cano, José López Miranda

**Affiliations:** 1 Medicine Faculty, Endocrine and Metabolic Diseases Research Center, The University of Zulia, Maracaibo, Venezuela; 2 Quantitative Methods Department, School of Economics, The University of Zulia, Maracaibo, Venezuela; 3 Lipid and Atherosclerosis Unit, Department of Medicine, IMIBIC/Reina Sofia University Hospital/University of Córdoba, Córdoba, Spain; 4 CIBER Fisiopatología Obesidad y Nutrición (CIBEROBN), Instituto de Salud Carlos III, Córdoba, Spain; University of Bremen, Germany

## Abstract

**Introduction:**

Obesity is a worldwide public health issue. Since the epidemiological behaviour of this disease is not well established in our country, the purpose of this study was to determinate its prevalence in the Maracaibo City, Zulia State- Venezuela.

**Materials and Methods:**

A cross-sectional study was undertaken using the data set from the Maracaibo City Metabolic Syndrome Prevalence Study. The sample consists of 2108 individuals from both genders and randomly selected: 1119 (53.09%) women and 989 (46.91%) men. The participants were interrogated for a complete clinical history and anthropometric measurements. To classify obesity, the WHO criteria for Body Mass Index (BMI), and Waist Circumference (WC) from the IDF/NHLBI/AHA/WHF/IAS/IASO-2009 (IDF-2009) and ATPIII statements were applied.

**Results:**

For BMI, obesity had an overall prevalence of 33.3% (n = 701), and according to gender women had 32.4% (n = 363) and men had 34.2% (n = 338). Overweight had a prevalence of 34.8% (n = 733), Normal weight had 29.8% (n = 629), and Underweight had 2.1% (n = 45). Adding Obesity and Overweight results, the prevalence of elevated BMI (>25 Kg/m^2^) was 68.1%. Using the IDF-2009 WC's cut-off, Obesity had 74.2% prevalence, compared to 51.7% using the ATPIII parameters.

**Conclusions:**

These results show a high prevalence of abdominal obesity in our locality defined by the WHO, IDF-2009 and ATPIII criteria, which were not designed for Latin-American populations. We suggest further investigation to estimate the proper values according to ethnicity, genetic background and sociocultural aspects.

## Introduction

The last five decades have beheld an alarming increase in obesity rates all over the industrialized world. Prevalence estimates of obesity usually are derived from surveys or population studies because systematic data on obesity generally cannot be gathered from medical records or vital statistics [1]–[2]. Virtually all data on prevalence and trends are derived on indirect body fat measurements based on weight and height (total adiposity) or using regional measures (plicometry) rather than on body fat because of the logistical difficulties involved in measuring body fat in population studies [1]–[2].

Obesity generally is defined as excess body fat but the definition of “excess”, however, is not clear-cut. Adiposity is a continuous variable not marked by a clear division between normal and abnormal. Moreover, it is difficult to measure body fat directly and consequently, obesity is often defined as excess body weight rather than as excess of fatness. Thus, in large epidemiologic and clinical studies, two basic approaches have been broadly used: The Body Mass Index calculation (BMI) and the waist circumference measurement (WC) [3].

The World Health Organization (WHO) categorizes obesity in classes according to the different cut-off points in BMI. Similar definitions were recommended by a National Heart, Lung, and Blood Institute (NHLBI) expert committee in the NHLBI Clinical Guidelines on the Identification, Evaluation, and Treatment of Overweight and Obesity in Adults [4]. On the other hand, abdominal circumference, albeit essential since it's a surrogate for visceral fat, has been subject to continued modification since central obesity values change according some factors like ethnicity and sex [5].

In Latin America, the epidemiology of obesity has not been studied in depth in most of the countries, but data from large studies from United States of America, Mexico and Brazil illustrate the magnitude of the problem in the Americas. In the United States, the latest NHANES [6] analyzed data from 2007–2008 in regards to obesity trend through 1999–2008, reporting that obesity is as high as 33.8% overall, 32.2% in men and 35.5% among women, being most prominent among non-Hispanic whites and blacks ethnics groups. In Mexico, the National Health and Nutrition Survey (2006) [7] revealed that central obesity is higher in women than in mean using ATPIII criteria, with 60.4% vs. 21.9% respectively. Obesity prevalence in the Mexican population has tripled over the last decade, from 9.8% to 39.3%, with especial concern in the poorer sectors of the economy [8] and adolescents [9]. As for Brazil, there was a 50% increase in obesity prevalence in men and women between 2002–2003, associated with similar frequencies in regards to overweight [10]. This tendency was observed among men in all socioeconomic strata, yet in women the trend was observed among the poorest women [10]. The VIGITEL survey reported that overweight prevalence in Brazil was 47% in men and 39% in women, while obesity was ∼11% for both sexes [11].

The epidemiological behavior of this disease is still unknown in the Maracaibo City (Zulia State-Venezuela), therefore we conducted an analysis of the data obtained in the “The Maracaibo City Metabolic Syndrome Prevalence Study” (MMSPS) [12] with the aim of laying the foundations to a broader initiative across our country to establish the epidemiological behavior of this condition among this population.

## Materials and Methods

### Ethics Statement

All participants signed a written consent before being interrogated and physically examined. The study was approved by the Ethics Committee of the Endocrine and Metabolic Diseases Research Center.

### Population Sample

The sample method was already published in the MMSPS cross-sectional proposal [12], yet the main aspects will be mentioned. Using population estimations for the population of Maracaibo (1,428,043 for 2007 according to the National Institute of Statistics), the sample size estimate was calculated to be 1,986 individuals with or above 18 years of age. Moreover, taking into account that in a previous pilot study approximately 10% of the subjects rejected being part of the study (unpublished data), an oversampling number was calculated (198 individuals); the overall number of patients was 2,108 (with 122 subjects −6.15% – added because of the oversampling method) were randomly selected between July 2008 and July 2010 [12]. The only inclusion criterion was to have ≥18 years of age. The city of Maracaibo is divided into parishes and each of these was proportionally sampled: Antonio Borjas Romero, Bolívar, Cacique Mara, Caracciolo Parra Pérez, Cecilio Acosta, Cristo de Aranza, Coquivacoa, Chiquinquirá, Francisco Eugenio Bustamante, Idelfonso Vásquez, Juana de Ávila, Luis Hurtado Higuera, Manuel Dagnino, Olegario Villalobos, Raúl Leoni, Santa Lucía, San Isidro, and Venancio Pulgar. The sampling process was undertaken using a 2-phase method: During the first phase, the sorting was random and stratified—where each stratus was represented by sectors from each of the 18 parishes—choosing 4 from each parish. The second sampling was stratified to represent a city block, in which they were selected using a random number generation tool. Once the houses were selected, every adult in the family unit from the selected city blocks was invited to participate in the study and were interviewed on prior written consent, and subjected to a routine medical examination using the clinical chart provided by the Health and Social Development Ministry of Venezuela as data collecting tool.

### Patient Evaluation

A full medical history was obtained using the Venezuelan Popular Powers Health Ministry approved medical chart. Socioeconomic Status was assessed with the Graffar Scale modified by Mendez-Castellano [13], which stratifies subjects into 5 Strata: High Class (Stratum I), Upper Middle Class, (Stratum II), Middle Class (Stratum III), Working Class (Stratum IV), and Lower - Extreme Poverty (Stratum V). In regards of race, the results will be presented as Hispanic whites, American-Indians (Natives), Afro-Venezuelans, Others (which include Arabic or Asian), and Mixed Race. Mixed race is a term applied to denote a group of individuals which have 2 or more (dihybrid or trihybrid) genetic lineages, denoting a complex interethnic crossing, which is a particular characteristic of the Latin American populations [14]–[15].

### Anthropometric Evaluation

#### Weight and Height measure and BMI Calculation

The WHO classification for Obesity is based upon the Body Mass Index formula [16] [Weight/Height^2^] expressed in Kg/m^2^. Height was obtained using a calibrated rod, millimeters and centimeters, with the patient barefooted and his/her back facing the wall. Weight was recorded using a digital scale (Tanita, TBF-310 GS Body Composition Analyzer, Tokyo – Japan) with the patient using light clothing and no shoes. The subjects are classified according to the following: Underweight below 18.50 Kg/m^2^, Normal Weight between 18.50–24,99 Kg/m^2^, Overweight (Pre-obese) between 25.00–29.99 Kg/m^2^, Obese Class I between 30.00–34.99 Kg/m^2^, Obese Class II between 35.00–39.99 Kg/m^2^, and Obesity Class III beyond 40.00 Kg/m^2^.

#### Waist Circumference

WC was measured using calibrated measuring tapes in millimeters and centimeters, using anatomical landmarks according to National Institutes of Health protocol [17]: midpoint between the lower border of the rib cage and the iliac crest, taking the length at the end of expiration, with participants standing and wearing only undergarments. The Adult Treatment Panel III (ATP III) [18] defined abdominal obesity according to Waist Circumference (WC), with a cut-off for men of 40 inches (102 cm) and 35 inches (89 cm) for women. The latest International Diabetes Federation/National Heart, Lung and Blood Institute/American Heart Association (IDF/NHLBI/AHA-2009) [19] consensus stated that WC was measured according to Country/Specific values, which for Latin Americans were set equal to South Asians parameters, specifically WC ≥90 cm for males and ≥80 cm for females. Since it was important to compare ATPIII and IDF/NHLBI/AHA criteria for abdominal obesity, we chose to apply the 2009 consensus cut-offs because the 2005 IDF criteria used the same values for abdominal circumpherence.

### Data Analysis

The data was analyzed by using the Statistical Package for the Social Sciences (SPSS) v. 19 for Windows (SPSS Inc. Chicago, IL). Normal distribution was evaluated by using Geary's test. For normally distributed variables the results were expressed as arithmetic mean ± SD (standard deviation), complemented with the Coefficient of Variation (CV). The differences between them were established using Student's *t*-test (when two groups were compared), analysis of variance (ANOVA), when three or more groups were compared, or proportions Z-test (when proportions between nominal variables were compared). The qualitative variables were expressed as absolute and relative frequencies, considering the results statistically significant when p<0.05. Age was analyzed only in a descriptive manner, being applied to stratify the population in decennial groups (grouping variable) and it was not used for other calculus. Since this was a randomized sampling cross-sectional study between 18 parishes, we compare the age pyramid obtained from the sample with the country's pyramid, obtaining the same characteristics: a young population which is common in Third World countries, which translates into a transition-type population [20]. Variability was verified using Levent test which rendered not significant except the 18–19 years old group because this decennial group is incomplete. To evaluate BMI, ANOVA was calculated for each gender taking into comparison the mean of each age group and using Tukey for post hoc testing.

## Results

### General Characteristics of the Population

The sample consisted of 2,108 individuals, of which 46.91% (n = 989) were males and 53.09% (n = 1119) were females. The average age was 38.68±15,42 years (CV = 42.04%), with a mean average of 36.97±14.94 years old (CV = 40.41%) for males and 40.19±15.67 years old (CV = 38.99%) for females. Stratifying this variable in decennial age groups showed that 8.6% of individuals (n = 181) were found in the group of 18–19 years, 27.6% of the individuals (n = 581) were in the group 20 to 29 years, 18% of the individuals (n = 380) were found in the group of 30–39 years, 20.1% of the individuals (n = 423) were found in the group of 40–49 years old, 15.2% (n = 320) were found in the 50–59 years group, 7.3% (n = 154) were from the 60–69 years group, and finally, 3.3% (n = 69) of the individuals were in the group of 70 or more years.

Analyzing the population by Ethnicity, the predominant group was of Mixed race with 75.23% (n = 1586), followed by Hispanic whites with 16.17% (n = 341). The remaining groups corresponded to American Indians (4.74%, n = 100), Afro-Venezuelans (3.13% n = 66), and Others (0.71%, n = 15). Evaluating the subjects according to socioeconomic status, the largest number of individuals were placed in Middle class with 838 members representing 39.75% of the total cases, followed by Lower Middle with 751 individuals (35,62%), Upper Middle class with 385 subjects (18.26%), Lower - Extreme poverty with 103 individuals (4.88%), and finally, Upper class with 31 individuals (1.47%).

### Obesity Prevalence according to BMI Classification

The BMI distribution matches with a normal distribution, which was confirmed by a Geary's test (p = 0.08). Mean BMI was 28.25±6.28 kg/m^2^ (95% CI 27.98–28.52, CV = 22.2%), with a minimum value of 14.22 kg/m^2^ and a maximum of 68.80 kg/m^2^.

**Figure 1:**
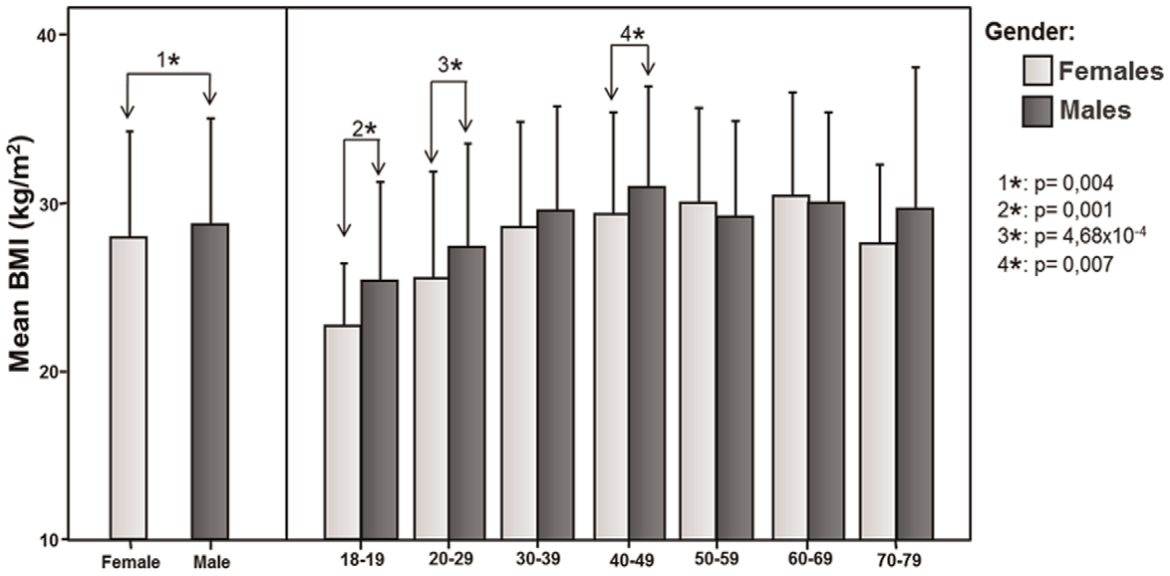
Body Mass Index according to age groups and gender, Maracaibo City 2010. The prevalence of Obesity was 32,4% for women and 34,2% for men. Significant difference (*) was observed between gender and in three age groups: ≤19 years, 20–29 years and 40–49 years.

The distribution between age groups, sex and ponderal classification according to BMI is depicted in Table 1 and Figure 1. The prevalence of Obesity (≥30 kg/m^2^) for both sexes and all ages was 33.3% (n = 701), distributed as follow: Obesity Class I 20.4% (n = 429), 8.7% (n = 183) for Obesity Class II, and 4.2% (n = 89) for Obesity Class III. The Underweight prevalence was only 2.1% (n = 45), meanwhile Normal-Weight was 29.8% (n = 629), and Overweight was 34.8% (n = 733). The prevalence of obesity on the female group was 32.4% (n = 363) with BMI of 27.87±6.30 kg/m^2^ (95% CI 27.50 to 28.24, CV = 22.6%). On the other hand, the prevalence of obesity for the male group was 34.2% (n = 338) with BMI of 28.68±6.25 kg/m^2^ (95% CI 28.29 to 29.07, CV = 21.8%); arithmetic mean difference was significant between both groups with p = 0.004. The BMI results according to age groups (Table 2) were analyzed using ANOVA (Table 3). Women's BMI rises steadily until the 30–39 years and 40–49 years where it stabilizes and progresses without significant difference. Table 2 shows the advancing gain of BMI, reaching a peak at 60–69 years, followed by a decline in the group ≥70 yrs old. On the other hand, males show a progressive BMI throughout time, which is significantly different with the youngest age group only.

**Table 1:** Distribution of the Population according to BMI, Sex and Age Groups, Maracaibo City 2010.

	Underweight		Normal Weight		Overweight		Obesity Class I		Obesity Class II		Obesity Class III		Obesity			
	<18,5 kg/m^2^		18,5–24,9 kg/m^2^		25–29,9 kg/m^2^		30–34,9 kg/m^2^		35–39,9 kg/m^2^		≥40 kg/m^2^		≥30 kg/m^2^		Total	
	n	%	n	%	n	%	n	%	n	%	n	%	n	%	n	%
***Females***	**30**	**2,7**	**378**	**33,8**	**349**	**31,2**	**215**	**19,2**	**99**	**8,8**	**49**	**4,4**	**363**	**32,4**	**1119**	**100**
**<20**	9	9	69	69	17	17	4	4	1	1	9	3,6	5	5,0	100	100
**20–29**	16	6,4	126	50,6	63	25,3	24	9,6	11	4,4	9	3,6	44	17,7	249	100
**30–39**	3	1,6	51	26,4	75	38,9	39	20,2	15	7,8	10	5,2	64	33,2	193	100
**40–49**	1	0,4	64	25,3	86	34,0	61	24,1	26	10,3	15	5,9	102	40,3	253	100
**50–59**	0	0	36	19,3	70	37,6	45	24,2	27	14,5	9	4,8	81	43,5	186	100
**60–69**	0	0	20	20,8	24	25,0	30	31,3	16	16,7	6	6,3	52	54,2	96	100
**≥70**	1	2,4	12	28,6	14	33,3	12	28,6	3	7,1	0	0	15	35,7	42	100
***Males***	**15**	**1,5**	**251**	**25,4**	**384**	**38,6**	**214**	**21,6**	**84**	**8,5**	**40**	**4,0**	**338**	**34,2**	**989**	**100**
**<20**	7	8,6	29	48,1	22	27,2	7	8,6	3	3,7	3	3,7	13	16.0	81	100
**20–29**	7	2,1	117	35,2	128	38,6	44	13,3	28	8,4	8	2,4	80	24,1	187	100
**30–39**	1	0,5	32	17,1	78	41,7	57	30,5	10	5,3	9	4,8	76	40,6	187	100
**40–49**	0	0	27	15,9	48	28,2	57	33,5	25	14,7	13	7,6	95	55,9	170	100
**50–59**	0	0	25	18,8	63	47	31	23,1	9	6,7	5	3,7	45	33,6	134	100
**60–69**	0	0	8	13,8	26	44,8	16	27,6	7	12,1	1	1,7	24	41,4	58	100
**≥70**	0	0	3	11,1	19	70,4	2	7,4	2	7,4	1	3,7	5	18,5	27	100
***Total***	**45**	**2,1**	**629**	**29,8**	**733**	**34,8**	**429**	**20,4**	**183**	**8,7**	**89**	**4,2**	**701**	**33,3**	**2108**	**100**

**Table 2:** Body Mass Index distributed according to Age Groups and Gender expressed as mean and standard deviation, Maracaibo City 2010.

	BMI (Kg/m^2^)			
	Females		Males	
Age Groups	Mean	SD	Mean	SD
**18–19**	22,61	3,73	25,28	5,87
**20–29**	25,54	6,28	27,36	6,10
**30–39**	28,53	6,23	29,51	6,21
**40–49**	29,31	6,05	30,91	5,97
**50–59**	29,93	5,65	29,22	5,61
**60–69**	30,42	6,11	30,02	5,30
**≥70**	27,58	4,66	29,65	8,34

**Table 3:** p values from ANOVA test when comparing BMI, according to Age Groups and Gender, Maracaibo City 2010.

	≤19 años	20–29 yrs	30–39 yrs	40–49 yrs	50–59 yrs	60–69 yrs	≥70 yrs
**<20 yrs**	-	0,081	3,67×10^−06^	1,93×10^−10^	8,64×10^−05^	1,19×10^−04^	0,020
**20–29 yrs**	0,001	-	0,002	1,45×10^−08^	0,044	0,034	0,486
**30–39 yrs**	4,02×10^−13^	2,44×10^−06^	-	0,301	1,000	0,998	1,000
**40–49 yrs**	3,89×10^−13^	2,26×10^−11^	0,813	-	0,192	0,960	0,952
**50–59 yrs**	3,89×10^−13^	8,01×10^−13^	0,230	0,924	-	0,981	1,000
**60–69 yrs**	3,89×10^−13^	1,50×10^−10^	0,136	0,696	0,995	-	1,000
**≥70 yrs**	8,98×10^−05^	0,360	0,963	0,569	0,219	0,122	-

The results shown here should be read as follows: the Female age groups are in the White boxes, while the Men age groups are in the Light Gray ones. Each Age group is compared as the table flows onwards. The black boxes represent the same age group which cannot compare with itself. ANOVA is Significant when p<0,05.

#### Obesity Prevalence adjusted by Ethnics Group and Socioeconomic status

Table 4 shows Obesity prevalence adjusted to each racial group and socioeconomic strata. According to ethnics, Mixed Race obtained 1,586 subjects (75,23%), of which 35% were Overweight, 29,6% were Normal-weight and an overall 33% of Obese (≥30 kg/m^2^) patients. Hispanic Whites group resulted in 341 individuals (16,17%), with 34,0% Overweight and an overall 37,8% Obesity. The Amerindian group attained 100 patients, with an overall 24,0% of Obesity and 33% Overweight. Finally, the Afro-Venezuelan group gathered 66 subjects, with an overall Obesity of 28,8% and 39,4% of Overweight. The proportions Z-test showed that there was no significant difference between the adjusted prevalences of obesity within each ethnic group.

**Table 4:** Distribution of the Population according to BMI, Ethnic Group and Socioeconomic Status, Maracaibo City 2010.

	Underweight		Normalweight		Overweight		Obesity Class I		Obesity Class II		Obesity Class III		Obesity			
	<18,5 kg/m^2^		18,5–24,9 kg/m^2^		25–29,9 kg/m^2^		30–34,9 kg/m^2^		35–39,9 kg/m^2^		≥40 kg/m^2^		≥30 kg/m^2^		Total	
	n	%	n	%	n	%	n	%	n	%	n	%	n	%	n	%
**Racial Group**																
**Mixed Race**	38	2,4	469	29,6	555	35	321	20,2	138	8,7	65	4,1	524	33,0	1586	100
**Hispanic White**	2	0,6	94	27,6	116	34	80	23,5	31	9,1	18	5,3	129	37,8	341	100
**Afro-Venezuelan**	2	3	19	28,8	26	39,4	9	13,6	8	12,1	2	3	19	28,8	66	100
**Amerindians**	3	3	40	40	33	33	15	15	5	5	4	4	24	24,0	10	100
**Others**	0	0	7	46,7	3	20	4	26,7	1	6,7	0	0	5	33,3	15	100
***Total***	**45**	**2,1**	**629**	**29,8**	**733**	**34,8**	**429**	**20,4**	**183**	**8,7**	**89**	**4,2**	**701**	**33,3**	**2108**	**100**
**Socioeconomic Status**																
**Stratum 1: High Class**	0	0	11	35,5	10	32,3	7	22,6	3	9,7	0	0	10	32,3	31	100
**Stratum II: Upper Middle Class**	2	0,5	127	33	130	33,8	72	18,7	35	9,2	19	4,9	126	32,7	385	100
**Stratum III: Middle Class**	23	2,7	234	27,9	298	35,6	163	19,5	75	8,9	45	5,4	283	33,8	838	100
**Stratum IV: Working Class**	18	2,4	219	29,2	268	35,7	162	21,6	64	8,5	20	2,7	246	32,8	751	100
**Sratum V: Extreme Poverty**	2	1,9	38	36,9	27	26,2	25	24,3	6	5,8	5	4,9	36	35,0	103	100
***Total***	**45**	**2,1**	**629**	**29,8**	**733**	**34,8**	**429**	**20,4**	**183**	**8,7**	**89**	**4,2**	**701**	**33,3**	**2108**	**100**

As for Socioeconomic status, Middle class prevailed with 838 individuals (39,75%), who obtained 35,6% Overweight and 33,8% of overall Obesity. Following, Lower Middle Class resulted in 751 subjects (33,62%), of which 35,7% were Overweight and an overall 32,8% were Obese. Upper Middle class attained 385 patients, with 35,7% Overweight and 32,7% Obese. It's noteworthy to mention that Extreme Poverty (Level V) obtained patients with Obesity Class I (n = 25, 24,3%), Obesity Class II (n = 6, 5,8%), and Obesity Class III (n = 5, 4,9%). As the previous paragraph, the adjusted prevalence of obesity within the socioeconomic strata showed no differences in the proportions Z-test.

### Abdominal Obesity According to IDF/NHLBI/AHA/WHF/IAS/IASO consensus (IDF-2009) and ATPIII criteria

Applying the IDF-2009 (Table 5) classification, Abdominal Obesity was prevalent in Females among the age groups of 60–69 years (94.8%), 50–59 years (94.7%), 40–49 years (89.3%), >70 years (88.1%) and 30–39 years (82.4%). Moreover, Abdominal Obesity frequency in the Male group was among 60–69 years (94.8%), >70 years (92.6%), 50–59 years (88.0%), 40–49 years (84.1%), and 30–39 years (79.7%). With the ATPIII cut-offs (Table 5), the incidence for Abdominal Obesity in the Female group was located in the >70 years (81.0%), 50–59 years (79.7%), 60–69 years (70.8%), 40–49 years (66.4%), and 30–39 years (61.7%); while, in the Male group, the most prevalent age group was 40–49 years (61.2%), 60–69 years (56.9%), 50–59 years (54.1%), >70 years (44.4%), and 30–39 years (38.0%).

**Table 5:** Distribution of the sample according to IDF2009 and ATPIII classification, Age Groups and Gender, Maracaibo City 2010.

	IDF2009				ATPIII			
	Obese		Normal		Obese		Normal	
WC	≥80 cms*≥90 cms&		≥80 cms*≥90 cms&		≥89 cms*≥102 cms&		≥89 cms*≥102 cms&	
***Females***	877	78,3%	243	21,7%	642	57,3%	478	42,7%
**<20**	36	36%	64	64%	13	13%	87	87%
**20–29**	151	60,6%	98	39,4%	91	36,5%	158	63,5%
**30–39**	159	82,4%	34	17,6%	119	61,7%	74	38,3%
**40–49**	226	89,3%	27	10,7%	168	66,4%	85	33,6%
**50–59**	117	94,7%	10	5,3%	149	79,7%	38	20,3%
**60–69**	91	94,8%	5	5,2%	68	70,8%	28	29,2%
**≥70**	37	88,1%	5	11,9%	34	81%	8	19%
***Males***	687	69,5%	301	30,5%	377	38,2%	611	61,8%
**<20**	26	32,1%	55	67,9%	11	13,6%	70	86,4%
**20–29**	172	51,8%	160	48,2%	74	22,3%	258	77,7%
**30–39**	149	79,7%	38	20,3%	71	38%	116	62%
**40–49**	143	84,1%	27	15,9	104	61,2%	66	38,8%
**50–59**	117	88%	16	12%	72	54,1%	61	45,9%
**60–69**	55	94,8%	3	5,2%	33	56,9%	25	43,1%
**≥70**	25	92,6%	2	7,4%	12	44,4%	15	55,6%
***Total***	**1565**	**74,2%**	**543**	**25,8%**	**1089**	**51,7%**	**1019**	**48,3%**

* Female's Waist Circumference cut-off.

& Male's Waist Circumference cut-off.

Table 6 shows mean and standard deviation of WC values according to age group. In the female group, a steady rise is observed throughout the tears, with a sudden decline at 70 years and beyond. On the other hand, the male group showed peaks at 40–49 years and 60–69 years, not sharing the steady climb seen in their counterparts. ANOVA was applied to each gender group to analyze WC behavior throughout time (Table 7). In the Females, the age group 20–29 years has significant differences between the rests of the decennial groups. Also, difference was observed between groups 30–39 years and 50–59/60–69 years. A similar tendency was observed in the male group, with the <20 years group having significant difference with the rest of the groups, while the 30–39 years only has significant differences with the groups below it and 40–49 years.

**Table 6:** Waist Circumference distributed according to Age Groups and Gender expressed as mean and standard deviation, Maracaibo City 2010.

	Waist Circumference (cm)			
	Females		Males	
Age Groups	Mean	SD	Mean	SD
**<20**	77,45	9,76	87,40	15,90
**20–29**	85,11	13,61	92,15	14,57
**30–39**	92,23	13,48	99,82	14,90
**40–49**	94,04	13,64	105,33	15,16
**50–59**	96,75	11,14	103,11	14,38
**60–69**	97,68	12,78	105,60	13,81
**≥70**	95,00	11,80	100,50	8,99

**Table 7:** p values from ANOVA test when comparing Waist Circumference means, according to Age Groups and Gender, Maracaibo City 2010.

	<20 yrs	20–29 yrs	30–39 yrs	40–49 yrs	50–59 yrs	60–69 yrs	≥70 yrs
**<20 yrs**	-	0,123	6,23×10^−09^	2,86×10^−13^	1,71×10^−12^	2,24×10^−11^	0,001
**20–29 yrs**	9,74×10^−06^	-	2,99×10^−07^	2,87×10^−13^	1,43×10^−11^	3,73×10^−09^	0,068
**30–39 yrs**	3,89×10^−13^	1,65×10^−07^	-	0,007	0,428	0,119	1,000
**40–49 yrs**	3,89×10^−13^	6,15×10^−13^	0,755	-	0,847	1,000	0,689
**50–59 yrs**	3,89×10^−13^	3,89×10^−13^	0,010	0,294	-	0,934	0,980
**60–69 yrs**	3,89×10^−13^	4,04×10^−13^	0,012	0,209	0,997	-	0,748
**≥70 yrs**	3,55×10^−12^	7,67×10^−05^	0,862	0,999	0,985	0,918	-

The results shown here should be read as follows: the Female age groups are in the White boxes, while the Men age groups are in the Light Gray ones. Each Age group is compared as the table flows onwards. The black boxes represent the same age group which cannot compare with itself. ANOVA is Significant when p<0,05.

**Figure 2:**
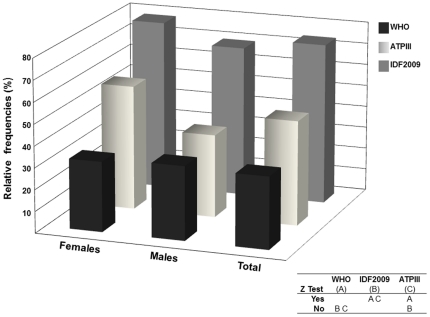
Prevalence of Obesity according to WHO, ATPIII and IDF-2009 criteria, Maracaibo City 2010. Using BMI (WHO) as tool for diagnosis, Obesity was observed in 33% of the sample and in both sexes. Taking waist circumference as reference, ATPIII obtained 51,7% of Obesity, in contrast to 74,2% applying IDF-2009's cut-offs. Using Z Test for proportions, significant differences were observed in between all classifications.

Using the WC classification from the IDF-2009, Abdominal Obesity prevalence was 74.2% (n = 1565), compared to 51.7% (n = 1089) using the ATPIII cut-offs. Even though Overweight and Obesity (≥30 Kg/m^2^) definitions from BMI criteria are two different categories, they convey information concerning adiposity that allows them to be used as surrogates for adipose disorders. In this light, combining Overweight and Obesity results show a 68.1% prevalence, which mildly correlates with the prevalence obtained using the IDF-2009 criteria (Figure 2).

## Discussion

In Latin America, obesity prevalence have tripled in the last 20 years, affecting adults and children throughout the continent [21], being nutritional transition the proposed cause of such phenomena [22]–[23]. Due to the advancing pace of economics, science, education, politics and other sociocultural topics, the current tendency is the emigration towards industrialized countries, or in this case, to urbanized areas. A large number of these emigrating families are characterized by a common nutritional deterioration owed to a change in their eating habits induced by the socio-cultural influence of the location [24]. Other factors influence this scenario like the reduction of breastfeeding, physical inactivity and easier access to mass media communication which promotes the acquisition of food that at first glance looks more attractive, but it is in fact more expensive and less nutritious [24]–[25].

In Venezuela, few studies have been conducted to estimate the burden, and those that are published might fail to be reliable due to lack of a proper sample size and statistical analysis; nevertheless since they are current information in regards to the county's situation. In 2003, Campo et al. [26] used 347 subjects to conduct a prevalence study, reporting obesity values in 74% of the men and 56.7% of the women, associating this state with dyslipidemia, hyperinsulinemia, and higher risk for cardiovascular disease. Nuñez et al. [27] evaluated the nutritional status of 360 subjects, reporting that 29.8% of the female and 23.3% of the male subjects were obese, classified as 65.79% in obesity type I, 23.68% as type II, and 10.52% as type III. Ryder et al. [28] published their results on metabolic syndrome components in a sample of 2,716 subjects, reporting that over half of the Hispanic blacks of their study were obese.

The prevalence of Overweight was 34,8% while Obesity was 33,3%. According to ethnic groups, the adjusted Overweight prevalence was predominant in the Afro-Venezuelans (39,4%), followed by Mixed Race (35%) and Hispanic White (34%). Meanwhile, for overall Obesity (≥30 Kg/m^2^), Hispanic Whites prevailed with 37,8%, followed by Mixed Race (33%) and Afro-Venezuelans (28,8%). This disease is most prominent within young and contemporary adults, with 27,8% in the 20–29 age group, 20.1% in the 40–49 years of age and 18% in the 30–39 age group, a trend that is probably related to working status and physical inactivity.

One of the most interesting finding in regards to socioeconomic stratification is the fact that Obesity (class II and III) occurrence is observed in the Extreme Poverty class. Obesity, once considered a disease of the wealthy, is now considered a threat to the lower socioeconomic statuses [29], raising itself as one of the newest paradoxes in the 21^st^ century [30]. The “Poor” and the “Rich” obese do not share the same characteristics; in fact, specific features can be pointed out [31]–[32]. “The Poor” obese is subject to genometabolic rearrangement (thrifty genotype) which enables him to efficiently store fats when subject to food fluctuation (food insecurity), situation that is fairly common in lower socioeconomic strata) and is relieved by the tendency to eat highly caloric meals mostly rich in carbohydrates and fat [33]. Meanwhile, the “Rich” obese is known for its sedentary lifestyle, use of motorized transportation and the fair access to junk food [33]. Obesity in low socioeconomic statuses is a rising wave within the Latin American population, especially when poor countries emerge from it strained economic and political situations, and poverty masses migrate toward urban areas [34]–[35], and these results may very well bear witness to such phenomenon in our city.

In regards to the natural history of weight gain, it can be described using WC's or BMI's progression throughout the years. In the female group, BMI and WC augmented gradually until 30–39 yrs group, suggesting that weight gain is prone to occur during childbearing age due to hormonal changes during pregnancy and nursing, a phenomenon which has been observed in Afro-American women [36]. Also, there seems to be an association between physical activity, number of children, socioeconomic status and obesity, which is predominant in societies with nutritional transition like Venezuela [37]–[38]. Meanwhile, male weight gain behavior shows a different pattern. BMI and WC modifications starts very early, probably while entering adulthood, and shows a progressive increase till 40–49 yrs, where it declines slightly yet it climbs again at 60–69 yrs, to finally succumb after 70 yrs of age. These ups and downs could associated with alcohol intake [39] whose effects are severe are earlier if started at an early age, cessation of smoking [40], or the appearance of a cardiovascular event which lead to temporal weight loss [41]. It's worth mentioning that early weight as observed in this cohort is associated with high risk coronary death and myocardial infarction [42]. In both genders, there is a decline in BMI and WC which is usually explained by sarcopenia in senior adults [43]–[44], but it's important to highlight that muscle loss is worst in those patients who were or are currently obese during this period. Sarcopenic obesity in the elderly is considered a major health problem, because in spite of being stereotypically being considered non-frail adults, they have low relative muscle mass and lower muscle strength [45], which puts them at risk medical complications, including vertebrae and hip fractures.

Applying the IDF-2009 and ATPIII classifications, an increase in Obesity diagnosis was observed using the former WC values, which uses lower cut-offs compared to the latter because they are based on Asian data. Even though IDF-2009 recommends the Asian cut-offs due to lack of appropriate studies and consensus [19], such indication doesn't fit with the Hispanic physiognomy. Asian populations are recognized to have lower BMIs which were enough to suggest that they needed new BMI cut-off points to classify obesity [46] Moreover, they have higher body fat percentages, especially in the subcutaneous compartment making them prone to generalized obesity despite their non-obese BMIs [47]–[48], which could explained by their skeletal dimensions [49]. Asians tend to have shorter legs lengths, smaller WC, longer trunks lengths and less gynecoid fat mass compared to Hispanics [50]–[51]. It has been previously published [52]–[53] that WC is a better indicator for cardiovascular risk than BMI in Hispanics, which only enhances the need for appropriate WC reference values in our population.

This study is part of the Maracaibo City Metabolic Syndrome Prevalence Study, a major research study to discover metabolic syndrome's prevalence, risk factors, and related comorbidities. In our country, two recent trials have assessed the associated risk between obesity and cardiovascular disease. In the first trial by Ruiz-Fernández et al. [54], the most prevalent cardiometabolic factors were low HDL, obesity/overweight, abdominal obesity, hypercholesterolemia, and insulin resistance. In the second trial, Espinoza et al. [55] reported that cardiovascular risk is strongly related to abdominal obesity, especially those with hypertriglyceridemic waist. Both of them used small samples (less than 100 patients) which makes them inappropriate for large population risk predictions.

Our study used a very representative sample from the Maracaibo City population, reporting a high prevalence of obesity using the WHO classification criteria for BMI and the WC's criteria from IDF-2009 and ATPIII. Nonetheless, these cut-off values are not set for ethnic groups like the ones represented in our population sample, which only highlights the need for proper ethnic specific criteria when anthropometric variables are applied. On a final note, it's noteworthy to mention that this study doesn't use self-reported or telephone questionnaires which allows for an accurate assessment of weight and height, without the inherent risk of overestimating or underestimating either of them respectively.

## References

[1] Seidell J, Flegal K (1997). Assessing obesity: classification and epidemiology.. Bri Medical Bulletin.

[2] Burkhauser R, Cawley J (2008). Beyond BMI: the value of more accurate measures of fatness and obesity in social science research.. J Health Economics.

[3] Centers for Disease Control Fact Sheet March 2003.. http://www.who.int/mediacentre/factsheets/fs273/en/.

[4] National Institutes of Health (1998). Clinical guidelines on the identification, evaluation, and treatment of overweight and obesity in adults—the evidence.. Obes Res Suppl.

[5] Misra A, Wasir J, Vikram N (2005). Waist circumference criteria for the diagnosis of abdominal obesity are not applicable uniformly to all populations and ethnic groups.. Nutrition.

[6] Flegal K, Carroll M, Ogden C, Curtin LR (2010). Prevalence and trends of obesity among US adults 1999–2008.. JAMA.

[7] Rojas R, Aguilar-Salinas C, Jiménez-Corona A, Shamah-Levy T, Rauda J (2010). Metabolic syndrome in Mexican adults. Results from the National Health and Nutrition Survey 2006.. Salud Publica Mex.

[8] Neufeld LM, Hernández-Cordero S, Fernald LC, Ramakrishnan U (2008). Overweight and Obesity doubled over a 6 year period in young women living in poverty in Mexico.. Obesity.

[9] Velasco-Martínez RM, Jiménez-Cruz A, Higuera-Dominguez F, Domínguez de la Piedra E, Bacardí-Gascón M (2009). Obesidad y resistencia a la insulina en adolescentes de Chiapas.. Nutr Hosp.

[10] Instituto Brasileiro de Geografi a e Estatística (2004). Pesquisa de orçamentos familiares 2002–2003..

[11] Gigante DP, Catarina de Mourai E, Sardinha LM (2009). Prevalence of overweight and Obesity and associated factors, Brazil, 2006.. Rev Saúde Pública.

[12] Bermúdez V, Marcano RP, Cano C, Arráiz N, Amell A (2010). The Maracaibo City Metabolic Syndrome Prevalence Study: Design and Scope.. Am J Ther.

[13] Méndez Castellano H, de Méndez MC (1986). Estratificación social y biología humana: método Graffar modificado.. Arch Ven Pueric Pediatr.

[14] Sans M (2000). Admixture studies in Latin America: from the 20th to the 21st century.. Hum Biol.

[15] Salzano FM (2004). Interthnic variability and admixture in Latin America – social implications.. Rev Biol Trop.

[16] World Health Organization The World Health Report 2003.. http://www.who.int/whr/2003/en/.

[17] Health Statistics. NHANES III reference manuals and reports (CDROM) (1996). http://www.cdc.gov/nchs/data/nhanes/nhanes3/cdrom/NCHS/MANUALS/ANTHRO.PDF.

[18] National Cholesterol Education Program (NCEP) Expert Panel on Detection, Evaluation, and Treatment of High Blood Cholesterol in Adults (Adult Treatment Panel III) (2002). Third Report of the National Cholesterol Education Program (NCEP) Expert Panel on Detection, Evaluation, and Treatment of High Blood Cholesterol in Adults (Adult Treatment Panel III) final report”.. Circulation.

[19] Alberti K, Eckecl R, Grundy S, Zimmet PZ, Cleeman JI (2009). Harmonizing the Metabolic Syndrome: A Joint Interim Statement of the International Diabetes Federation Task Force on Epidemiology and Prevention: National Heart, Lung, and Blood Institute; American Heart Association; World Heart Federation; International Atherosclerosis Society; International Association for the Study of Obesity.. Circulation.

[20] Omran AR (1996). The epidemiologic transition in the Americas.

[21] Uauy R, Albala C, Kain J (2001). Obesity Trends in Latin America: Transiting from Under- to Overweight.. J Nutr.

[22] Ibañez L (2007). El Problema de la Obesidad en América.. Rev Chil Cir.

[23] Bernstein A (2008). Emerging patterns in overweight and obesity in Ecuador.. Rev Panam Salud Publica.

[24] Pérez B (2010). http://www.slan.org.ve/publicaciones/completas/efecto_urbanizacio_salud_poblacion.asp.

[25] Díaz J, Cagigas R (2005). Hábitos Alimentarios y Estado Nutricional de Centroamérica y el Caribe. Instituto de Nutrición e Higiene de los Alimentos.. http://www.inha.sld.cu/Documentos/habitos_alimentarios.pdf.

[26] Campos G, Ryder E (2003). Prevalencia de obesidad e hiperinsulinemia en una población aparentemente sana de Maracaibo, Venezuela y su relación con las concentraciones de lípidos y lipoproteínas del suero.. Invest Clín.

[27] Nuñez R, Peña A (2006). Obesidad en pacientes adultos del Municipio Sucre del Estado Miranda.. Archivos Venezolanos Farmacología Terapéutica.

[28] Ryder E, Silva E, Sulbaran T, Fernández V, Campos G (2007). Los Hispanos negros tienen un perfil de riesgo cardiovascular peor que los hispanos mezclados en Venezuela”.. Invest Clín.

[29] Raymond S (2006). Obesity and cardiovascular disease in developing countries: a growing problem and an economic threat.. Curr Opin Clin Nutr Metab Care.

[30] Tanumihardjo S, Anderson C, Kaufer-Horwitz M, Bode L, Emenaker NJ (2007). Poverty, Obesity, and malnutrition: an international perspective recognizing the paradox.. J Am Diet Assoc.

[31] Peña M, Bacallao J (2000). Obesity among the Poor: an emerging problem in Latin American and the Caribbean.. PAHO.

[32] Drewnowski A, Specter SE (2004). Poverty and Obesity: the role of energy density and energy costs.. Am J Clin Nutr.

[33] Kuczmarski R, Flegal KM, Campbell SM, Johnson CL (1994). Increasing prevalence of overweight among US adults: The National Health and Nutrition Examination Surveys, 1960 to 1991.. JAMA.

[34] Uauy R, Albala C, Kain J (2001). “Obesity trends in Latin America: Transiting from Under to Overweight.. J Nutrition.

[35] Rosenberg L, Palmer JR, Wise L, Nicholas JH, Shiriki KK (2003). A propective study of the effect of childbearing age on weight gain in African-American women.. Obesity Res.

[36] McLaren L (2007). Socioeconomic status and obesity.. Epidemiology Rev.

[37] Kain J, Vio F, Albala C (2003). Obesity trends and determinant factors in Latin America.. Cad Saúde Pública.

[38] Bermúdez OI, Tucker KL (2003). Trends in dietary patterns of Latin American populations.. Cad Saúde Pública.

[39] Wannamethee SG, Sharper SG (2003). Alcohol, body weight, and weight gain in middle-aged men.. Am J Clin Nutr.

[40] Williamson DF, Madans J, Anda RF, Kleinman JC, Giovino GA (1991). Smoking cessation and severity of weight gain in a National cohort.. NEJM.

[41] Ades PA (2001). Cardiac rehabilitation and secondary prevention of Coronary Heart Disease.. NEJM.

[42] Rosengren HW, Wilhelmsen L (1999). Body weight and weight gain during adult life in men in relation to coronary heart disease and mortality.. Eur Heart J.

[43] Baumgatner R, Stauber PM, McHugh D, Koehler KM, Garry PJ (1995). Cross-sectional age differences in body composition in persons 60+ years of age.. J Gerontol A Biol Sci Med Sci.

[44] Roubenoff R, Hughes VA (2000). Sarcopenia.. J Gerontol A Biol Sci Med Sci.

[45] Villareal DT, Banks M, Siener C, Sinacore DR, Klein S (2004). Physical frailty and body composition in obese elderly men and women.. Obesity Res.

[46] Banerji MA, Faridi N, Atluri R, Chaiken RL, Lebovitz HE (1999). Body composition, visceral fat, leptin, and insulin resistance in Asian Indian Men.. J Clin Endocrinol Metab.

[47] Raji A, Seely EW, Arky R, Simonson D (2001). Body fat distribution and insulin resistance in healthy Asian Indians and Caucasians.. J Clin Endocrinol Metab.

[48] Wildman RP, Gu D, Reynolds K, Duan X, He J (2004). Appropriate body mass index and waist circumference cutoffs for categorization of overweight and central adiposity among Chinese adults.. Am J Clin Nutr.

[49] Novotny R, Going S, Teegarden D, Van Loan M, McCabe L (2007). Hispanic and Asian pubertal girls have higher android/gynoid fat ratio than Whites.. Obesity.

[50] Malina RM, Huang YC, Brown KH (1995). Subcutaneous adipose tissue distribution in adolescent girls of four ethnic groups.. Int J Obesity.

[51] Zhu S, Heymsfield SB, Toyoshima H, Wang Z, Pietrobelli A (2005). Race-ethnicity-specific waist circumference cutoffs for identifying cardiovascular disease risk factors.. Am J Clin Nutr.

[52] Okosun IS, Liao Y, Rotimi CN, Prewitt TE, Cooper RS (2000). Abdominal adiposity and clustering of multiple metabolic syndrome in White, Black and Hispanic Americans.. Ann Epidemiol.

[53] Okosun IS, Liao Y, Rotimi CN, Choi S, Cooper RS (2000). Predictive values of waist circumference for dyslipidemia, type 2 diabetes and hypertension in overweight White, Black and Hispanic American adults.. J Clin Epidemiol.

[54] Ruiz-Fernández N, Espinoza M, Barrios E, Reigosa A (2009). Cardiometabolic factors in a community located at Valencia City, Venezuela.. Rev Salud Publica (Bogota).

[55] Espinoza ZM, Ruiz FN, Barrios E, Reigosa A, Leal HU (2009). Cardiovascular risk profile and insulin resistance according body mass index, waist circumference and hypertriglyceridemic waist in adult subjects.. Rev Med Chil.

